# The first abdominal aortic aneurysm organoid model replicates complex microenvironment for *in vitro* disease study

**DOI:** 10.7150/thno.118193

**Published:** 2025-08-16

**Authors:** Jiaxuan Feng, Mingjie Rong, Yudong Sun, Guanglang Zhu, Guangkuo Wang, Jiping Liu, Chen Wang, Jian Zhang, Xiaochen Ma, Junyi Yan, Yaojie Wang, Youjin Li, Yu Ning, Chunhui Cai, Xinxin Han

**Affiliations:** 1 Department of Vascular Surgery, Department of General Surgery, Ruijin Hospital, Affiliated to Shanghai Jiao Tong University School of Medicine, Shanghai, 200025, China.; 2 Shanghai Lisheng Biotech, Shanghai, 200092, China.; 3 Department of Cardiovascular Surgery, Jiangmen Central Hospital, Guangdong, 529000, China.; 4 Department of Cardiovascular Surgery, People's Hospital of Ningxia Hui Autonomous Region, Ningxia, 750002, China.; 5 Department of Vascular Surgery, The First People's Hospital of Yulin, Guangxi, 537000, China.; 6 LiSheng Organ Regeneration X Lab, East China Institute of Biotechnology, Peking University, Jiangsu, 226299, China.

**Keywords:** Abdominal aortic aneurysm, Organoid, Patient-derived organoid, Immune microenvironment, *In vitro* disease model, Cardiovascular disease

## Abstract

**Background:** Abdominal Aortic Aneurysm (AAA) is a critical global health issue, affecting an estimated up to 8% of men over 65, with a complex etiology involving smoking, age and gender. However, the lack of specific drug treatments underscores the need for a robust in vitro model to advance our comprehension of AAA pathophysiology and serve as an ex vivo surrogate for drug testing.

**Methods:** This study introduces an innovative AAA patient-derived organoid (PDO) model using a non-enzymatic procedure, a Matrigel-free system, and specialized organoid culture medium, leveraging 3-dimensional (3D) cultures to replicate the disease's microenvironment. The stability of this culture system was assessed through microscopic observation, H&E staining, immunohistochemistry (IHC), viability assays, and whole-genome sequencing (WES). Additionally, we conducted pharmacological assessments to explore the effects of drug treatments on AAA PDO.

**Results:** Our model maintains aortic morphological integrity and pathological phenotypes, incorporates the immune microenvironment (validated by IHC markers for macrophages and lymphocytes), and adjacent tissues (loose connective tissue and vegetative blood vessels). The model demonstrates remarkable stability, confirmed by consistent genetic mutation sites throughout cultivation via WES, and cell survival after five weeks in vitro via live-cell staining. Preliminary pharmacological assessments show promising efficacy, with distinct responses to 1 μM metformin, 1 μM RU.521, or 1 μM STING-IN-2 treatments for 48 h via mass spectrometry.

**Conclusions:** The AAA organoid model, to the best of our knowledge, is the first reported abdominal aortic aneurysm PDO model, and signifies a promising step towards therapeutic treatment options for AAA, potentially complementing existing surgical approaches.

## Introduction

Abdominal aortic aneurysm (AAA) is a critical and prevalent vascular disorder characterized by the abnormal enlargement of the abdominal aorta to more than 3 cm in diameter, typically asymptomatic and potentially leading to rupture and life-threatening hemorrhage 1, 2. The global impact of AAA is substantial, contributing to over 1% of all mortalities among men aged 65-85 in developed countries 1. A study of over 3 million individuals revealed that patients aged 55 and above accounted for a significant proportion (>95%) of AAA cases across various sizes (3-6 cm) 3. A complex interplay of hereditary and environmental risk factors contributes to AAA, most notably older age, smoking, male sex, and a positive family history 2, 4-8.

The pathological basis of AAA lies in the loss of vascular structural integrity. This process includes the degradation of the extracellular matrix (ECM) and apoptosis of vascular smooth muscle cells (VSMCs) 9, 10. Inflammation and the infiltration of inflammatory cells are key factors contributing to the disease's progression through the release of reactive oxygen species (ROS), which induce oxidative stress within the aortic wall 11-20. Additionally, the secretion of pro-inflammatory cytokines such as tumor necrosis factor-alpha (TNF-α) and interleukin-6 (IL-6) further fuels inflammation and tissue damage. The action of proteolytic enzymes, such as matrix metalloproteinase-9 (MMP-9), secreted by inflammatory cells, weakens the structural integrity of the aortic wall and accelerates aneurysmal degeneration 11-13.

Current clinical management for high-risk AAA is primarily surgical, involving endovascular aortic repair and open surgical repair 13, 14. Despite the prevalence of AAA, there is a significant lack of an effective model and no pharmacological treatments with proven enough efficacy, highlighting the need for a deeper understanding of the disease mechanisms and the development of novel treatment strategies 21, 22. Human organoids, as innovative *in vitro* models, emulate the intricate microenvironment of human tissues and organs, offering a potential tool for studying organ development, function, disease mechanisms, modeling and drug screening 23, 24. Furthermore, human organoids are recognized as an ideal model in clinical and research settings for their potential to bridge the gap between traditional *in vitro* models and* in vivo* systems, offering a more physiologically relevant context for investigation 25. Their ability to recapitulate tissue-specific architecture and physiological conditions makes organoids a valuable tool for studying AAA pathogenesis and for identifying potential therapeutics.

In this study, we present a novel organoid model of AAA designed to emulate the *in vivo* 3D architecture and some functionalities of the abdominal aorta, serving as a biological ex vivo surrogate for drug testing and pathophysiological investigation. Previously, AAA models were mostly developed from traditional animal models by drug-induced vascular inflammatory responses 26-29. Efforts to develop functional vascular organoids have involved differentiating induced pluripotent stem cells into endothelial and vascular wall cells. However, these models did not specifically target AAA 30, 31. Some studies have utilized 3D printing technology to create cystic dilation structures of human vascularized tissue to simulate AAA, mainly focusing on biomechanical properties like hemodynamic effects on vascular dilation 32. Additionally, dispersing smooth muscle cells (SMCs) from AAA patients onto electrospun poly-lactide-co-glycolide scaffolds has been explored for creating AAA models with certain mechanical and biological characteristics being demonstrated 33. In contrast, our culture system encompasses a multifaceted microenvironment including elements of the immune microenvironment, *vasa vasorum,* perivascular connective tissue and the model's morphological consistency, preserved pathological phenotypes and genomic steadiness are well retained. Early pharmacological testing on the model has indicated its potential, with drug response data providing preliminary but positive indications. This makes it the first abdominal aortic aneurysm PDO model in the world. The innovative approach provides a potentially useful tool for advancing our understanding of AAA pathophysiology and facilitating the discovery of effective therapeutics.

## Results

### AAA PDO modeling and morphological consistency

**Figure 1:**
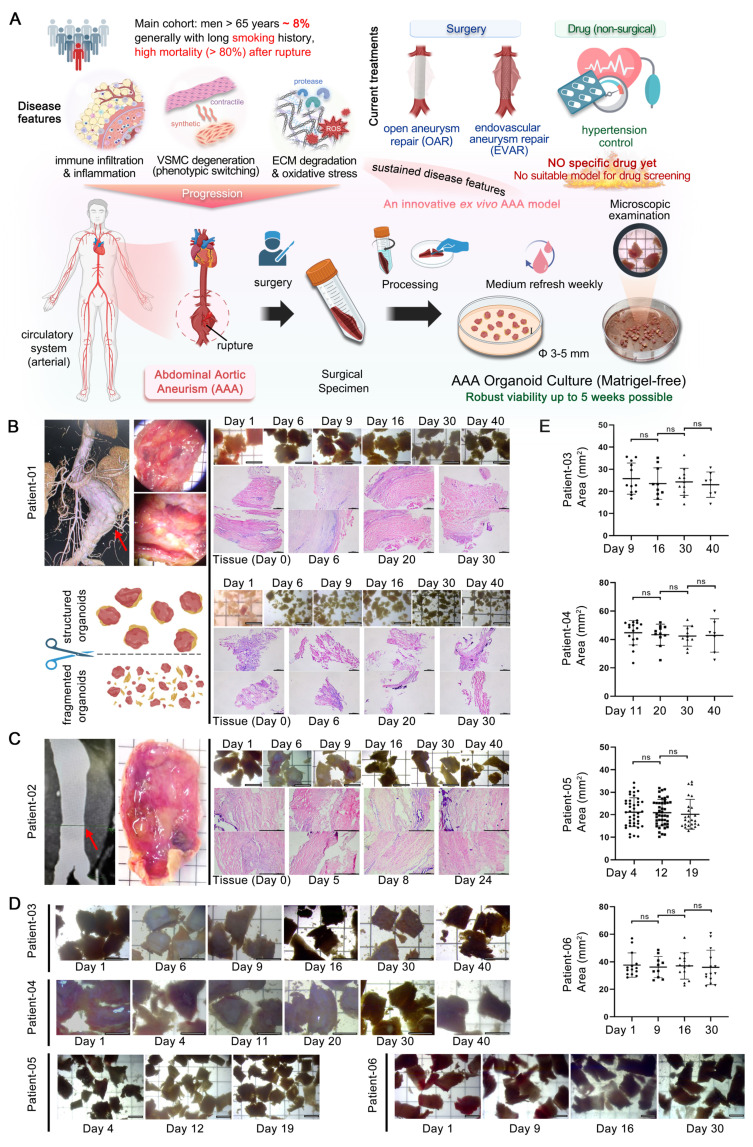
Modeling and Growth of AAA PDO. (A) Anatomical location of AAA, from clinical tissue specimens to organoid culture. (B) Tissue sampling location images for Patient 01, comparing two culture methods: preserved layered structure and minced (bright-field microscopy and H&E staining). (C) Tissue sampling location images for Patient 02. (D) Bright-field microscopy of the culture process for Patients 03-06. (E) Corresponding organoid size changes to Figure 1D, with size measurements completed using ImageJ. Variable sample size n = 7-44 due to sampling during cultivation. Bright-field imaging Scale Bar = 5 mm, H&E staining Scale Bar = 500 μm. Data are represented as the mean ± SEM, with individual data points displayed. Illustration and statistical analysis were performed using GraphPad 9.0.

To preserve the native microenvironment and anatomical structure of AAA tissues, we developed a method for generating AAA PDO without the need for enzymatic dissociation into single cells. Considering the substantial extracellular matrix of the aortic walls and the limited proliferative capacity, which poses challenges for enzymatic digestion and risks cell death with prolonged digestion times, enzymatic treatment was avoided. Furthermore, the AAA PDO models were established without the use of Matrigel or other biomaterials; they were cultured in a special medium without constraints. For details, AAA specimens were collected from patients post-surgery. Following thoroughly washing, the specimens were trimmed into rectangles measuring approximately 3-5 mm on each side using surgical scissors, and subsequently transferred to a 10 cm culture dish filled with 20 mL of culture medium (Figure 1A).

Regular sampling was conducted to evaluate morphological changes. Medical imaging data and stereomicroscope observations, as well as bright-field and hematoxylin & eosin (H&E) staining images of samples with different culture durations from Patient 01 and 02, are demonstrated in (Figure 1B-C). Additionally, a series of bright-field images from PDO derived from Patient 03 to 06 across varying days in culture are presented (Figure 1D). The size changes of these PDO were statistically analyzed (Figure 1E). No significant variations were observed throughout the culture period. Altogether, the AAA PDO exhibited no notable morphological changes under bright-field microscopy, indicating that their structural integrity was well maintained.

### The PDO model preserved AAA pathological phenotypes

To evaluate the similarity of AAA PDO to their native tissues, we performed histological analyses. The H&E staining indicated that sampling of fully minced organoid was non-systematic, potentially resulting in the acquisition of solely the loose connective tissue of the adventitia, and its structure appeared more dispersed relative to the intact tissue (Figure 1B). In contrast, the "rectangular" PDO were capable of preserving a dense, layered structure. Consequently, in subsequent culturing, all tissue processing ensured the preservation of the layered structure, avoiding fragmentation.

**Figure 2:**
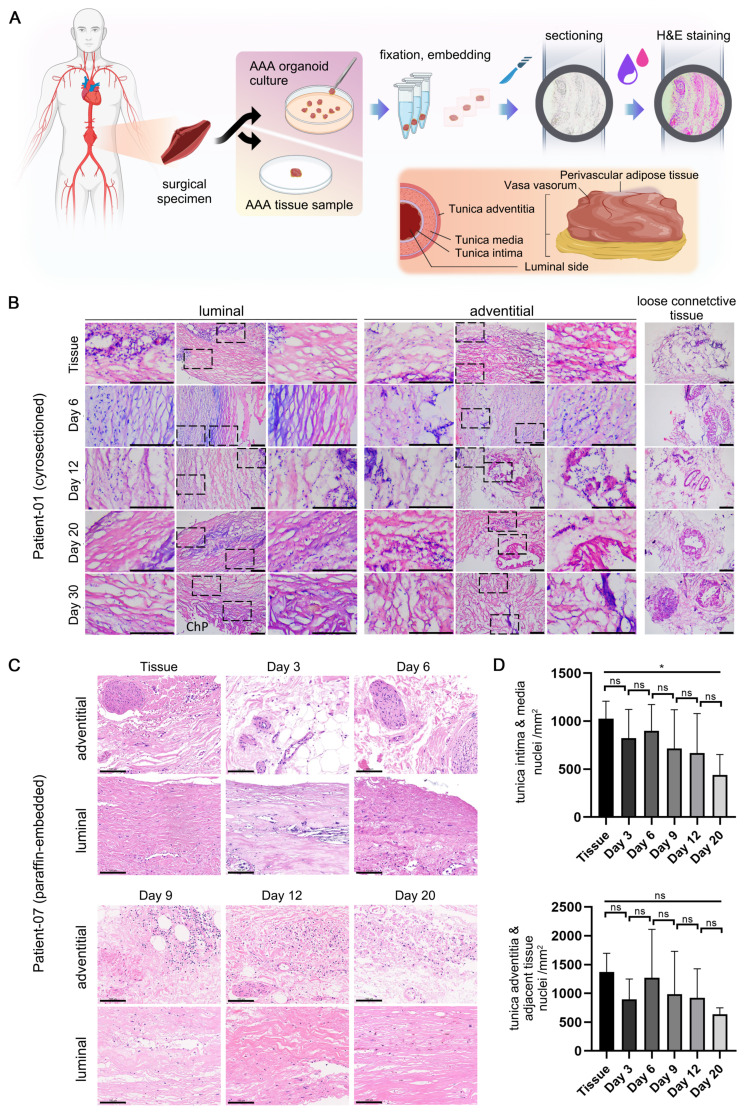
H&E Staining of AAA PDO. (A) Schematic diagram of the staining process and anatomical illustration of abdominal aortic tissue. (B) Cryosection H&E staining images from Patient 01. Scale Bar = 200 μm. ChP = Cholesterol Plaque (C) Paraffin section H&E staining images from Patient 07. Scale Bar = 100 μm (D) Quantitative analysis based on Figure 2C. Using ImageJ to calculate the number of cell nuclei per unit area (n = 6 ROIs). The Tunica intima & media area and the Tunica adventitia & adjacent tissue area were analyzed separately. Data are represented as the mean ± SEM. Illustration and statistical analysis were performed using GraphPad 9.0.

Detailed H&E staining data are organized into two principal sections (Figure 2A). The tissue and organoid samples from Patient 01, following cryosectioning and staining (Figure 2B), retained similarities with the tissue post 30 days in culture, exhibiting nuclei that were dispersed in a seemingly random pattern. The arrangement of the media, or luminal region, is denser and more uniformly structured than that of the adventitia. Additionally, loose connective tissues, including Perivascular Adipose Tissue (PVAT) and *vasa vasorum*, adjacent to the adventitia, are clearly discernible.

The tissue and organoid samples from Patient 07, following paraffin embedding, sectioning, staining (Figure 2C), and high-resolution scanning, produced results with enhanced clarity. The tissue morphology remained consistent throughout the culture process, with no notable loss of cells, particularly vascular smooth muscle cells, observed. According to the quantitative analysis (Figure 2D), the mean number of nuclei exhibited a slight decline with increasing culture duration up to Day 20. Furthermore, the adventitia, which is adjacent to loose connective tissue, contained a significantly higher cell count relative to the luminal side.

A key pathological feature of AAA is the degradation of the extracellular matrix (ECM), particularly characterized by the loss of elastic fibers, contributing to the risk of aneurysm dilation and rupture. Hence, it is essential to ascertain whether the culture system can preserve the native ECM structure of the tissue. Elastic staining, using the Verhoeff-van Gieson technique, was utilized (Figure S1A). The staining of cryosectioned samples (Figure S1B) demonstrated that elastic fibers in the tissue and PDO with diverse culture durations consistently presented a brief, filamentous (S-shaped) or truncated rod-like morphology, without any significant loss, even after a four-week culture period. By segmenting the stained images into distinct regions and conducting a quantitative analysis to determine the proportion of elastic fibers within the tissue, it was observed that the percentage of elastic fibers showed only a minimal reduction, if any, over extended culture periods (Figure S1C). Consequently, the culture system effectively maintains the physiological and pathological integrity of the elastic fibers.

### The AAA PDO owned sustained microenvironment

**Figure 3:**
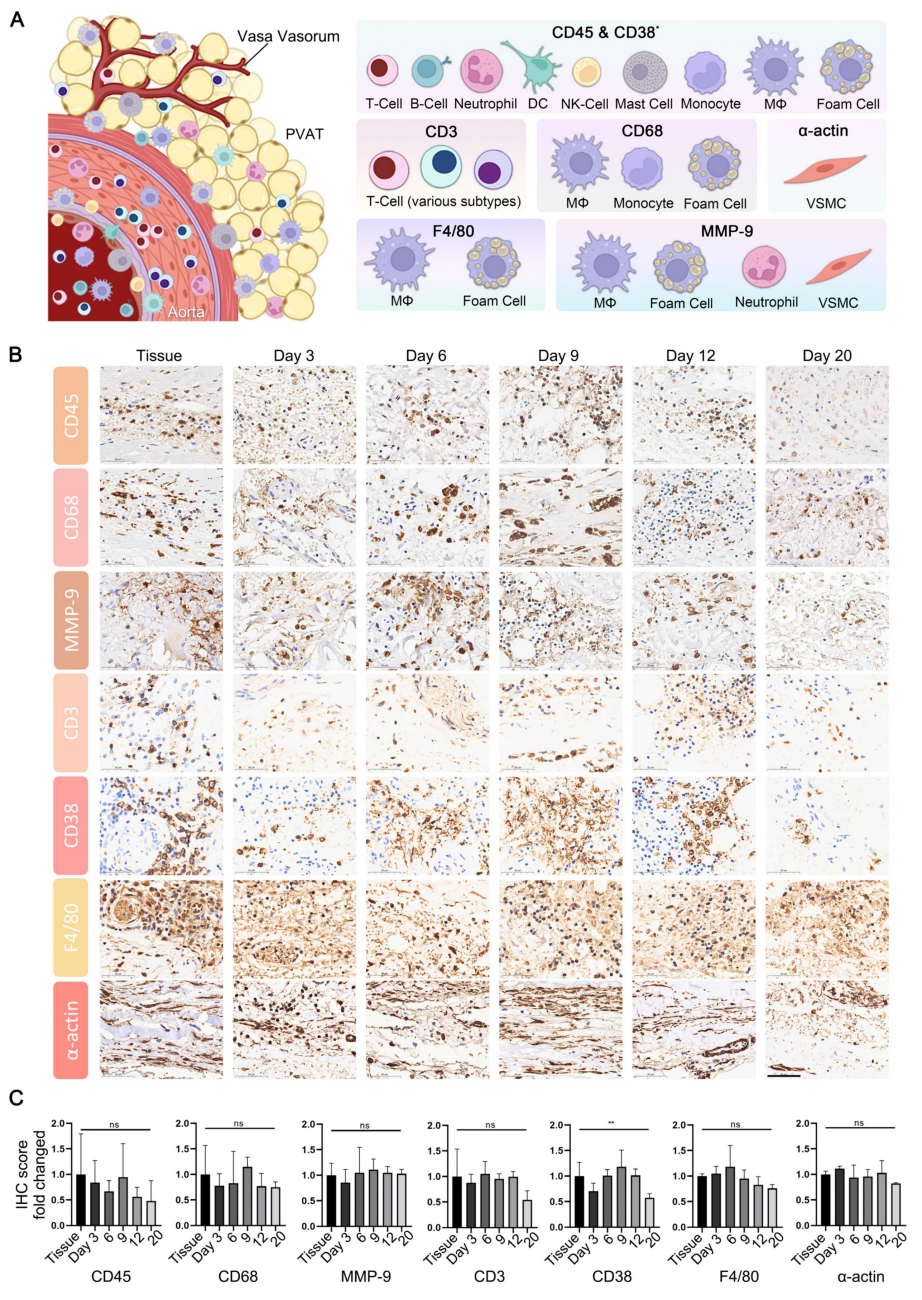
Characterization of Microenvironment in AAA PDO via Immunohistochemistry (IHC). (A) Schematic representation of the vascular immune microenvironment, along with the cells corresponding to various marker. DC = Dendritic Cell, NK = Natural Killer, MΦ = Macrophage, VSMC = Vascular Smooth Muscle Cell, PVAT = Perivascular Adipose Tissue. Created with BioRender.com and Adobe PhotoShop 2024. (B) IHC images of related markers. scale bar = 50 μm. (C) Quantitative analysis of IHC images: Original data were divided into four approximate equal parts with minimal overlap and blank space (< 20%). The ImageJ IHC profiler plugin was utilized to score the percentage of positive areas. The scores for high positive, positive, and low positive areas were multiplied by factors of 3, 2, and 1, respectively, and then summed. Data are represented as the mean ± SEM. Illustration and statistical analysis were performed using GraphPad 9.0.

In addition to ECM degradation, key pathological characteristics of AAA encompass extensive inflammatory infiltration by immune cells and the degeneration of smooth muscle. The effective preservation of the immune microenvironment is a critical benchmark for successful organoid culture, thereby enhancing the comprehension of the disease process and facilitating drug target assessment. Immunohistochemistry (IHC) is a technique that effectively and conveniently delineates the immune microenvironment. We selected a panel of immune cell markers, including CD3, CD38, CD45, CD68, F4/80, and MMP-9 for testing. Additionally, α-actin was utilized to assess the capacity of the culture system to preserve the native state of smooth muscle in tissue (Figure 3A).

The expression profiles of CD3, CD38, CD45, CD68, F4/80, MMP-9, and α-actin in both tissue and PDO are demonstrated, with specific staining for these markers showing moderate attenuation as the organoid culture period extends (Figure 3B, Figure S2). For a quantitative assessment of these results, the tissue sections were manually segmented into four distinct areas, ensuring minimal overlap and blank space, with both maintained below 20%. Subsequent quantitative analysis was conducted using the IHC profiler plugin in ImageJ, scoring the positive areas. Statistical analysis reveals no significant differences in the expression of CD3, CD45, CD68, MMP-9, F4/80 and α-actin between tissues and PDO across various culture time points (Figure 3C). However, CD38 showed significant variation due to uneven distribution at different time points, as determined by one-way ANOVA analysis. From an overall trend perspective, the expression of the aforementioned markers in organoid cultures remains relatively stable within the first 12 days.

### The genomic steadiness of AAA organoid

**Figure 4:**
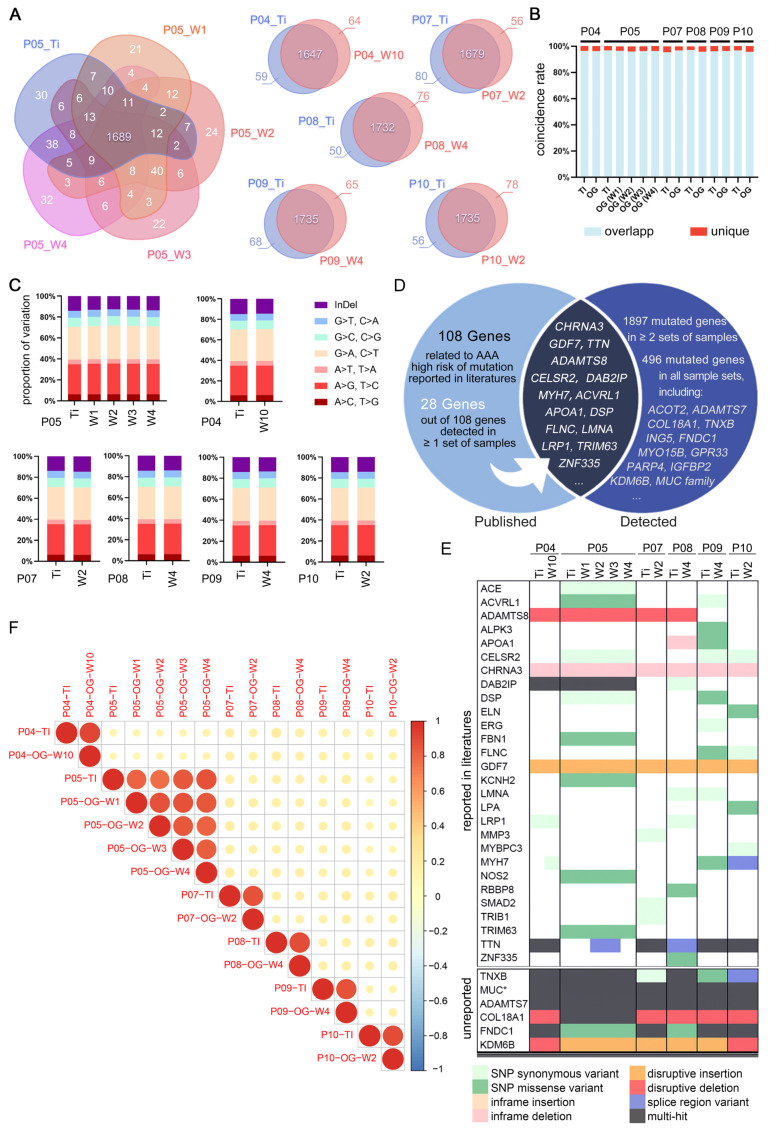
WES Analysis Confirming Genomic Stability. (A) Venn diagram of mutations in tissue and corresponding organoids, highlighting commonalities and differences. (B) Coincidence rate derived from Figure 4A. (C) Proportional distribution of InDels and point mutations. (D) Venn diagram comparing high-frequency mutations found in this study to those reported in literature. (E) Overview of gene mutation types, with the upper section depicting literature-consistent mutations and the lower section listing high-risk mutation genes detected, not clearly reported but associated with cardiovascular diseases. ^*^MUC family including MUC1/2/3A/4/5AC/6/12/16/19/20/21/22. (F) Correlation of mutation frequencies between tissue and organoids samples. Pearson r values are ≥ 0.75 for all within-patient pairs, indicating strong concordance. TI = Tissue, OG = Organoids, W = week (organoid culture duration), P = patient.

Through WES, we confirmed the genomic stability of AAA organoids, showing high genetic similarity to source tissues (Figure 4A). The concordance in mutation profiles between tissue and organoid samples was notably high, exceeding 95% (Figure 4B). The proportions of insertions and deletions (InDels) and single nucleotide polymorphisms (SNPs) were highly consistent, further confirming the genetic stability of our culture system (Figure 4C).

Cross-sample analysis revealed many shared mutation sites. We identified 108 risk genes frequently mutated in AAA patients from literatures 34-38, 28 of which were confirmed by our samples, including notably recurrent genes such as ADAMTS8, CELSR2, CHRNA3, DAB2IP, GDF7, MYH7, and TTN (Figure 4D). The specific mutations are shown in Figure 4E.

WES also detected numerous previously unreported high-frequency mutation sites, enhancing our understanding of AAA's genetic basis. We found 496 mutated genes present in both tissues and organoids across all six patients. Some of these genes are strongly associated with cardiovascular diseases, such as the MUC family, COL18A1, and TNXB. Various mutation types and frequencies were observed within these genes (Figure 4E). Within each patient-matched sample group, the variant allele frequency between matched tissue and organoid was highly concordant (Figure 4F), indicating that the organoid model did not markedly alter the overall mutation burden within the analyzed exome.

### The organoid viability maintenance during cultivation

Maintaining viability over a specified duration is a critical criterion for the successful establishment of AAA organoid models and essential for their subsequent application in drug testing. Following the initial development of the models, the PDO maintained roughly 50% of their initial viability after approximately 12 days in culture.

**Figure 5:**
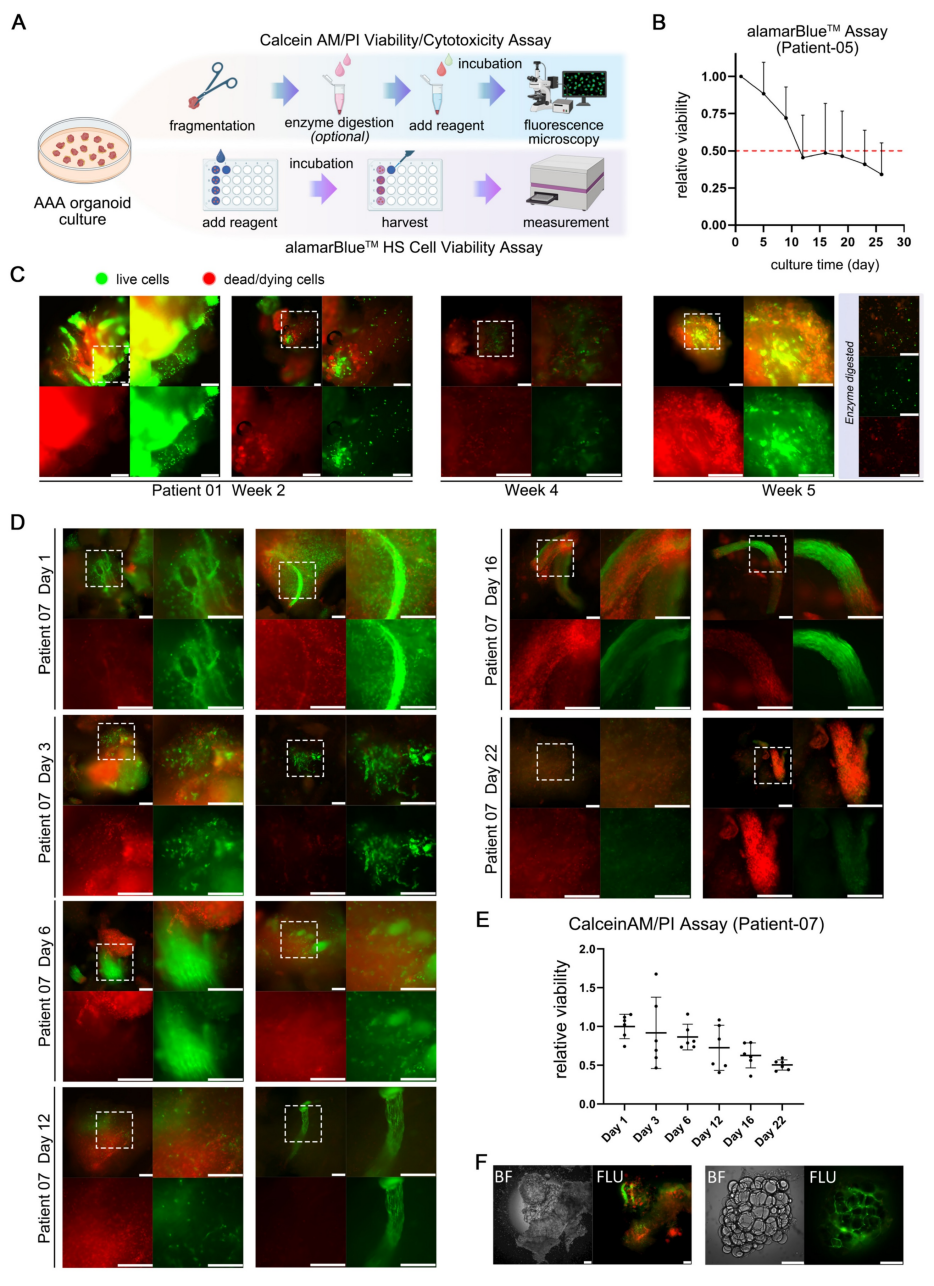
Viability Assessment of AAA PDO. (A) Schematic diagrams of fluorescence-based viability assays. (B) Results of the alamarBlue™ HS assay. Fluorescence units, after background subtraction, were normalized to the values from the initial test (Day 1-2) to calculate the fold change. Data are represented as the mean ± SEM. Illustration was created with GraphPad 9.0. (C, D) Results of the Calcein AM/PI assay for fragmented AAA PDO. Green fluorescence indicates live cells, red fluorescence signifies dead or dying cells. Scale bar = 200 μm. (E) Quantitative analysis of the images from Figure 6C. Images were converted to 8-bit grayscale in ImageJ, and the mean fluorescence intensity of red and green channels was measured after applied a constant threshold, respectively. Subsequently, the ratio of green to red fluorescence intensity was calculated, and then normalized to the arithmetic mean of values on Day 1. Data are represented as the mean ± SEM, with individual data points displayed. Illustration was created with GraphPad 9.0. (F) Fluorescent stained perivascular connective tissue from AAA organoid fragments. BF = Bright-field image; FLU = Merged red/green fluorescence image after Calcein AM/PI staining. Scale bar = 200 μm.

This study utilized two distinct fluorescence-based assays for the assessment of viability (Figure 5A). The alamarBlue™ HS assay, employing a non-fluorescent blue dye, offers an alternative approach. The dye is directly incorporated into the culture medium and subjected to a 20 h incubation period, during which it is converted to a strong red fluorescent product by viable cells. Owing to its low toxicity, this assay facilitates the continuous monitoring of viability changes within the same sample over time. PDO derived from Patient 05 were concurrently tested for viability using this assay (Figure 5B), exhibiting an average viability decline to 50% of the initial state after approximately 12 days of culture.

The Calcein AM/PI assay entails the direct fluorescent staining of viable cells, characterized by green fluorescence, in contrast to non-viable cells that exhibit red fluorescence. The staining patterns of fragmented PDO derived from two individual patients at multiple culture time points are illustrated in (Figure 5C-D). PDO derived from Patient 01 exhibited sustained robust viability even after 5 weeks, characterized by intense punctate green fluorescence upon enzymatic dissociation, signifying the presence of metabolically active cells. Nonetheless, the samples from the fourth week demonstrated a modest decrease in viability relative to the fifth week, possibly attributable to sampling variability, batch effects, or human error. The AAA PDO derived from Patient 07 underwent more frequent sampling and staining, initially showing high viability on Day 1 and 3, which subsequently gradually declined, as confirmed by quantitative analysis (Figure 5E). In addition, Calcein AM/PI staining effectively demonstrates the culture system's ability to maintain complex tissue structure including loose connective tissue, as seen in the green fluorescent stained *vaso vasorum* and the adipose tissue in (Figure 5F).

### Organoid responses to drug treatments

**Figure 6:**
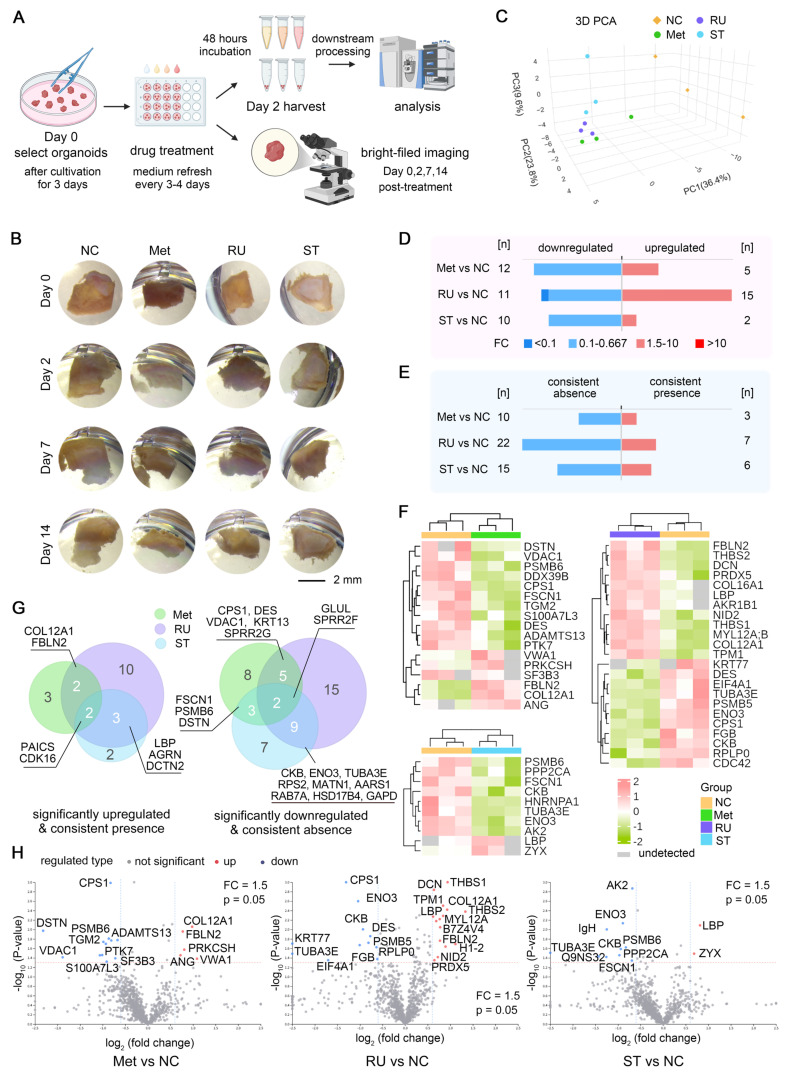
Drug Test in AAA PDO. (A) Schematic representation of the drug treatment process, with subsequent bright-field imaging and analysis of supernatants by mass spectrometry. (B) Bright-field images captured on days 0, 2, 7, and 14. (C) 3D PCA plot of the four test groups. (D) Number of differentially expressed proteins, with emphasis on up- and downregulation. (E) Number of differentially expressed proteins, emphasizing consistent presence and absence across samples. (F) Heatmap of differentially expressed proteins post-hierarchical clustering, highlighting up- and downregulation. (G, H) Venn diagram and Volcano Plot illustrating the proteins significantly upregulated/emerged and downregulated/lost in different drug treatment groups compared to the control, highlighting commonalities and differences. (H) NC = negative control (solvent control 0.01% DMSO), Met = Metformin (1 μM, with 0.01% DMSO), RU = RU.521 (1 μM, with 0.01% DMSO), ST = STING-IN-2 (1 μM, with 0.01% DMSO); FC = fold change.

The preliminary evaluation of pharmacological interventions within our AAA organoid model showcased its great potential as a therapeutic tool and approach for AAA treatment. Following the validation of the stability of this organoid culture system, we commenced pharmacological treatment of AAA PDO derived from samples of Patient 10 on the third day of cultivation with 1 μM RU.521 (RU), 1 μM STING-IN-2 (ST), accompanied by a solvent control and another control of 1 μM metformin (Met), which has the potential for the treatment of AAA 22, 39, 40. After 48 h of incubation, the supernatants were subjected to mass spectrometry analysis. Conversely, the bright-field images were captured on days 0, 2, 7 and 14 post-treatments (Figure 6A). Bright-field imaging (Figure 6B) shows that after 14 days of drug treatment, the AAA PDO did not exhibit notable morphological changes. In the PCA analysis (Figure 6C), samples within each group were closely clustered, with distinct differences observed between the drug-treated groups and the control group, indicating satisfactory sample quality.

In comparison to the control groups, each drug treatment group displayed a number of proteins with markedly distinct expression profiles. A comparative analysis of the supernatants from each drug treatment group versus the control group is presented, highlighting proteins that are upregulated, downregulated, or show consistent presence or absence, respectively (Figure 6D-E). Moreover, a clustered heatmap of the proteins significantly upregulated or downregulated is provided, allowing for the determination of their distribution, differences, and clustering relationships (Figure 6F). The Venn diagram and Volcano plot (Figure 6G-H) illustrate the proteins that are significantly upregulated or newly emerged and downregulated or lost in different drug treatment groups compared to the control, highlighting both commonalities and differences. This suggests that different drug treatments could show similar effects on the expression of certain proteins. Furthermore, the impact of the RU.521 on the protein expression pattern is noticeably more pronounced than that of the other two drugs.

## Discussion

The development of AAA organoid model represents a significant advancement in vascular disease research, offering a physiologically relevant platform for AAA studies. Our innovative model, engineered to replicate the intricate microenvironment of AAA, has undergone validation for multifaceted stability and has exhibited promising efficacy in initial pharmacological assessments.

Currently, the predominant focus of organoid research is on tumor-derived organoids 41. Subsequently, considerable interest lies in organoids induced by stem cells and those derived from somatic cells or tissues with proliferative potential 42, 43. Numerous diseases including AAA encounter limitations in developing *ex vivo* models due to their restricted proliferative capacity and the complexity of their pathology 44. The prevalent method of investigation is currently animal-based, exemplified by the use of murine models of AAA. However, they are labor-intensive, expensive, and may not accurately reflect the pathophysiological mechanisms observed in humans 25. Furthermore, the disparity between promising preclinical results and the absence of corresponding success in clinical trials may indicate inherent limitations in the current models for aneurysm disease representation 21. Hence, there is a pressing demand for the development of a dependable human-derived* in vitro* AAA model.

At present, the primary method for organoid cultivation involves enzymatic dissociation into single cells, subsequent encapsulation with Matrigel 45, or the application of technologies such as microfluidics or the air-liquid interface (ALI) culture system 46. Our cultivation system is notably innovative, offering convenience by allowing unrestricted cultivation in a standard culture dish using a specialized culture medium, thereby eliminating the requirement for enzymatic dissociation 47, or Matrigel 48, 49. This organoid cultivation approach, by significantly retaining tissue characteristics and the microenvironment, provides a more precise reflection of the authentic physiological and pathological states *in vivo* 48-50.

The progression of AAA disease is predominantly driven by inflammation, extracellular matrix (ECM) degradation, and apoptosis of vascular smooth muscle cells (VSMCs) 51, 52. Employing this culture system, the AAA PDO have demonstrated the structural and pathophysiological features characteristic of AAA. We have established the stability of this culture system through the following criteria: Throughout a month-long cultivation period, the PDO maintained sustained integrity, exhibiting no signs of degradation or morphological changes. The cellular and ECM phenotype was confirmed using H&E and elastic staining, and the preservation of the immune microenvironment along with the morphology of smooth muscle cells was validated through immunohistochemistry.

On the other hand, the genome of PDO has proven to be remarkably stable, showing no aberrant mutations introduced by* in vitro* cultivation, thereby validating the model's reliability and highlighting its suitability for drug exposure studies. In the present study, the samples were sourced exclusively from elderly male patients with a documented history of chronic smoking, within the age range of 61 to 73 years. Although this demographic represents a principal group at risk for AAA, additional risk factors encompass familial genetic conditions, hypertension, hyperlipidemia, and atherosclerosis. Notably, a smoking history, male gender, advanced age, and genetic predisposition are recognized as significant risk factors 53. Consequently, genomic analysis is considered to be of considerable importance in this context. We have compiled a comprehensive list of 108 genes from extant literature that are correlated with a high risk of mutation in AAA 34-38. A substantial overlap was noted among 28 of these genes identified in our whole-exome sequencing (WES) analysis of tissue and organoid samples from six individuals. Prominent among these overlapping genes are growth differentiation factor 7 (GDF-7) 34, a disintegrin and metalloproteinase with thrombospondin type 1 motif 8 (ADAMTS8) 35, cadherin EGF LAG seven-pass G-type receptor 2 (CELSR2) 36, Disabled homolog 2 (DAB2IP) 37, cholinergic receptor nicotinic alpha 3 (CHRNA3) 34, titin (TTN) 38, and myosin heavy chain 7 (MYH7) 38, among others. Additionally, we identified 496 mutated genes across all tissue and organoid samples, including those associated with cardiovascular diseases, such as mucin MUC family members 54, collagen type XVIII alpha 1 (COL18A1) 55, ADAMTS7 56, tenascin XB (TNXB) 57, fibronectin type III domain containing 1 (FNDC1) 58, and lysine demethylase 6B (KDM6B, also known as JMJD3) 59. The majority of these genes displayed multiple mutations, indicating a sophisticated genetic landscape in the etiology of AAA. This comprehensive genetic analysis highlights the potential for a refined comprehension of the disease and could facilitate the development of targeted therapeutic strategies.

Preliminary drug testing using our AAA organoid model has produced encouraging outcomes, indicating its potential for identifying candidate therapeutic agents. In this study, we evaluated the effects of RU.521 and STING-IN-2, utilizing metformin as a positive control. Metformin, a member of the biguanide class, is well-known for its glucose-lowering effect with high safety. In addition to its action on glucose metabolism, the drug has demonstrated effects on the circulatory system and has shown participation in other biological pathways, including ECM remodeling, VSMC homeostasis, oxidative stress response, and anti-inflammatory activity 22. It has also been associated with a reduced risk of AAA development 22, 40. RU.521 is a small-molecule inhibitor targeting the cGAS/STING/NF-κB pathway, exerting anti-inflammatory effects 60. Research indicates that it selectively suppresses cGAS-mediated signaling, leading to reduced interferon expression in macrophages within rodent models 61. Likewise, STING-IN-2 is a selective covalent small molecule inhibitor that has been validated for its potent inhibitory activity against the stimulator of interferon genes (STING) protein, resulting attenuated STING-mediated inflammatory cytokine production in both human and murine cells 62.

Proteomic analysis of the supernatant from organoid cultures following drug treatment indicated that this organoid model could elicit distinct or partially overlapping responses to different drugs. Interpreting from a molecular perspective (Figure 6F-G), the expression of fibulin-2 (FBLN-2) was notably upregulated in groups treated with metformin or RU.521. FBLN-2 directs the assembly of elastic fibers and is responsible for the maintenance of the vessel wall post-injury 63. The upregulation of collagen type XII α1 chain (COL12A1) may contribute to the remodeling of ECM 64. Decorin (DCN), another ECM protein, regulates ECM assembly, fibrillogenesis, and biological processes, including acting as a transforming growth factor-β regulator 65, 66. The upregulated expression of lipopolysaccharide-binding protein (LBP) in the RU.521- and STING-IN-2-treated groups may be associated with lipid droplet homeostasis and resistance to oxidative stress 67. Thrombospondin-1 and -2 (THBS-1 and -2), uniquely upregulated in the RU.521-treated group, are implicated in vascular development, function, and exhibit anti-inflammatory activities 68. Angiogenin (ANG), related to angiogenesis and ribonucleolytic activity, was uniquely upregulated in the metformin-treated group 69. Zyxin (ZYX), a cytoskeletal and focal adhesion scaffold-related protein, modulates angiogenesis and cytoskeleton enlargement, was uniquely upregulated in the STING-IN-2-treated group 70. Certain proteins, absent in the control group, emerged in drug-treated groups; for instance, agrin (AGRN), which is involved in the neuromuscular junction, inflammatory suppression and angiogenesis 71, 72, as well as cyclin-dependent kinase 16 (CDK16), which is regulated by the AMPK pathway and implicated in autophagy 73. Conversely, several proteins were significantly downregulated in the drug-treated groups. For example, destrin (DSTN), desmin (DES), fascin (FSCN1), Ras-related protein Rab-7a (RAB7A), glutamine synthetase (GLUL) and multiple ribosomal subunits were markedly downregulated in at least two drug treated groups. Destrin and desmin have been identified as potential factors involved in aneurysm formation 74-76. Fascin, which plays a role in the cross-linking of actin filaments 77, could potentially affect vascular smooth muscle cells and influence the integrity and function of the arterial wall. Rab-7a, involved in endocytosis and autophagy, regulates the phenotypic transformation and behavior of vascular smooth muscle cells in human aortic dissection 78. Glutamine synthetase is implicated in angiogenesis, serving functions beyond glutamine synthesis 79. Furthermore, the cell division cycle protein (CDC42) was specifically downregulated in the group treated with RU.521. This protein is associated with the recruitment and activation of immune cells 80, 81, potentially contributing to inflammation in AAA. Upon comparison of the list of significantly altered protein expressions with public databases, LBP, DEX, ZYX, THBS-1, and CDC42 were identified as proteins related to immune pathways. Although the underlying mechanisms necessitate additional clarification, these significant findings underscore the organoid culture system's potential for drug screening and other applications.

Despite our promising results, the study is accompanied by several limitations. Due to difficulties in sample collection, our subjects were exclusively elderly male smokers. Ideally, our sample pool should encompass a more diverse demographic, including individuals with familial genetic predispositions associated with AAA and other relevant factors, as well as those without any known risk factors. In the future, we aspire to enhance our biobank to incorporate a broader range of samples 82. Data obtained from viability assays indicate a progressive decline in organoid viability over extended culture periods. Consequently, we opted to conduct drug testing at an earlier stage, specifically within the initial week of cultivation. While accelerated cultivation and drug response may enhance research efficiency, future refinements must prioritize the maintenance of viability over prolonged periods, and culture methodologies should be standardized. For instance, to preserve the stratified structure of the vascular wall, efforts should be directed toward reducing the size of individual PDO to achieve greater uniformity. This approach would facilitate improved material exchange and metabolism, enhance utilization rates, and potentially increase the sample size. In the PCA analysis, a small number of proteins with different expression levels were derived solely from the supernatant, limiting further pathway studies. In the future, we intend to optimize our experimental design to attain a more effective and reproducible PCA.

Further research should consider the incorporation of more complex systems, such as Organoids-on-a-Chip 83, entailing the arrangement of the aortic wall's diverse structural layers, including perivascular adipose tissue (PVAT) and thrombi, within distinct compartments. Alternatively, microfluidic technology could be utilized to replicate blood flow and implement vacuum or pressure changes to emulate blood pressure 84. Such advanced models could provide a more physiologically relevant context for studying the behavior of organoids and their responses to pharmacological treatments.

## Conclusion

In conclusion, our AAA organoid model encapsulates a notable progression in the field, providing an innovative platform for the investigation of AAA pathophysiology and evaluating potential treatments. Characterized by its capacity to replicate key disease characteristics and maintain stability throughout an elongated timeframe, this model will be well-suited for pharmaceutical screening. As an ex vivo surrogate of the human body, it offers a unique opportunity to study the intricacies of AAA in a controlled *in vitro* microenvironment. As we further refine this model for extended applications, it is poised to augment our comprehension of AAA and contribute to the discovery of effective therapeutic strategies.

## Experimental Section/Methods

### Sample preparation

Aortic tissue specimens were obtained from 11 AAA patients (ages 61 to 73 years, mean age 67.2 ± 3.3 years, Table 1) who underwent resection of AAA with prosthetic graft replacement at Jiangmen Central Hospital, Guangdong Province, China. Samples were stored in a sterile vessel without tissue storage solution and transported to Shanghai LiSheng Biotech (Shanghai, China) within 24 h. The clinical samples were obtained in accordance with the ethical guidelines of Jiangmen Central Hospital and were granted institutional ethics approval with the number 2024-77 A. All procedures performed in studies involving human participants were in accordance with the ethical standards of the institutional and/or national research committee and with the 1964 Helsinki Declaration and its later amendments or comparable ethical standards. Informed consent was obtained from all individual participants included in the study.

### Sample processing and organoid culture

The tissue specimens were immersed in a washing buffer (LSTO01200201; Shanghai LiSheng Biotech, China) to preliminarily remove blood and then transferred to a 50 mL centrifuge tube (430829; Corning, USA) for three rounds of washing with the same buffer. Each round involved the addition of 5 mL of washing buffer and gentle agitation to rinse the tissue surface, followed by a 3 min wait. After removing the supernatant from the final round, the tissue blocks were then transferred to a 10 cm culture dish (430167; Corning, USA). The tissue blocks were sectioned into rectangles with sides measuring approximately 3-5 mm using surgical scissors and then transferred to a new 10 cm culture dish. A small portion of the tissue was reserved for sectioning and pathological staining, while another portion was retained for whole exome sequencing. Finally, 20 mL of culture medium was added to the dish (LSTO01200403; Shanghai LiSheng Biotech, China) and the cultures were maintained at 37 ℃, 5% CO_2_.

Throughout the organoid cultivation, partial medium replenishment is essential, typically every 5-7 days or based on the medium's visual characteristics, including color and turbidity, to maintain the required nutrient levels for organoid metabolism. The technique for partial medium exchange involves tilting the culture dish to facilitate the removal of 8-10 mL of the old medium from the upper layer using a serological pipette. Then, 10-12 mL of freshly prepared medium was added, with an additional 1-2 mL of medium also considered to compensate for potential volume reduction due to evaporation during the cultivation.

### PDO size calculation and analysis

The PDO were routinely imaged, and their sizes were quantified using ImageJ. Using a dissecting microscope with a relatively broad field of view (YZ38; Shanghai YueHe Biotech, China), the area of each organoid was calculated to track changes in size over time. Considering the limited proliferative capacity of AAA PDO, which is a distinction from the classic "spheroid" organoids, their shape, determined by the tissue processing technique, was often polygonal or rectangular. Consequently, the area analysis was conducted in ImageJ by manually tracing the organoid's perimeter.

### Whole exome sequencing (WES)

For each sample, 200 ng genomic DNA was fragmented to 150-200 bp to construct libraries. Subsequently, the whole exome was captured using AIExome^®^ Human Exome Panel V3 and TargetSeq One^®^ Hyb & Wash Kit v2.0 (iGeneTech Co., Ltd, Beijing, China) and sequenced on DNBSEQ-T7 with 150-bp reads.

### Fixation, embedding and sectioning

Routine collection of samples was performed, followed by immersion in a PBS solution with 4% paraformaldehyde (PFA) (BL539A; Biosharp, China) for 48 h fixation. Subsequently, the samples were subjected to a gradient dehydration process using a PBS solution with sucrose at concentrations of 10%, 20%, and 30% (A15583.0E; Thermo Fisher Scientific, USA) for 1 h, 2 h, and overnight, respectively. Following encapsulation of the organoids in an optimal cutting temperature compound (Tissue-Tek® O.C.T. Compound, 4583; Sakura Finetek, Japan), the samples were immediately immersed in liquid nitrogen to form embedding blocks for cryosectioning. The embedding blocks were then subjected to cryosection at -20 °C (Cryostat CM1950; Leica, Germany), with sections cut perpendicular to the vessel's lumen to expose the stratified layers of the aortic wall. Alternatively, a subset of samples was processed for paraffin embedding and sectioning. Once the fixation reagent was removed, the samples were enveloped in 2% molten agarose. After cooling and solidification, the regions containing the samples were excised, and the specimens were subjected to a dehydration (TP1020; Leica, Germany) and paraffin infiltration series as follows: 75% ethanol for 4 h, 85% ethanol for 2 h, 90% ethanol for 2 h, 95% ethanol for 1 h, 100% ethanol for 30 min (repeated three times), xylene for 5-10 min (twice), and 65 °C molten paraffin for 1 h (three times). Subsequently, the samples were embedded using an embedding machine (KD-BM IV; KEDEE, China), and the paraffin blocks were trimmed to a size conducive to sectioning (RM2016; Leica, Germany) after cooling at -20 °C.

### H&E staining

Cryosectioned slides were fixed with 4% PFA in PBS for 10 min and subsequently rinsed in water. The slides were then stained with Hematoxylin and Eosin (H&E) (C0105S; Beyotime, China), beginning with a 10 min application of hematoxylin, followed by rinsing in water for 10 min. The sections were differentiated using acid alcohol (C0163M; Beyotime, China) for 5 s. Following this, the slides were rinsed again in water for 10 min and counterstained with eosin for 30 s to visualize the cytoplasm and extracellular matrix. The slides were dehydrated through a graded alcohol series and mounted with neutral balsam (Type D, G8593; Solarbio, China). Examination of the stained slides was performed using a standard microscope (YI21; Shanghai YueHe Biotech, China). On the other hand, paraffin sections necessitate an additional deparaffinization procedure prior to staining. The slides were sequentially immersed in the following solutions: xylene for 20 min (twice), 100% ethanol for 5 min (twice), and 75% ethanol for 5 min, rinse in water. After staining, the sections were dehydrated with 100% ethanol and clarified with xylene for 5 min. Following this, the slides were mounted with neutral balsam and prepared for scanning (KF-DPS-120; Kfbio, China).

For quantitative analysis based on H&E staining, ImageJ was utilized, and the ratios were calculated as the number of cell nuclei per area of the section. Images were processed using the Color Deconvolution plugin in ImageJ with the H&E Vectors to separate Hematoxylin-stained components. Subsequently, a threshold of 1-180 was applied, fine-tuning as necessary to clearly display cell nuclei without severe overlap and minimal background noise. Anatomically corresponding Regions of Interest (ROIs) were selected; six non-overlapping ROIs were selected in total, each measuring 600 μm^2^ in size. Application of the Analyze Particles function was then made to each ROI with a size range of 5-Infinity μm^2^ for counting. For statistical analysis, GraphPad 9.0 software was employed, and a t-test was performed to compare tissue to PDO.

### Verhoeff-van Gieson elastic staining

Cryosectioned slides were fixed in 4% PFA in PBS for 10 min, then rinsed in water for 10 min. The slides were subsequently stained with a mixture of 5% alcoholic hematoxylin (HE-013-100mL; BIOISCO, China), 10% ferric chloride (Iron(III) chloride, anhydrous, A600454-0500; Sangon Biotech, China), and Lugol's Iodine Solution (MM1049-100mL; MKBio, China) in a 1:1:0.5 ratio for 15 min. Following a 1 min rinse in water, slides were differentiated with 2% ferric chloride, then rinsed again for 1 min. Then, the slides were soaked in 5% sodium thiosulfate (217263-5G; Sigma-Aldrich, USA), rinsed in water for 1 min, and soaked in Van Gieson's Stain (MM1032; MKBio, China) for 30 s. After staining, the slides were dehydrated through a series of 100% alcohol and mounted using neutral balsam. The slides were examined using a standard microscope.

Quantitative analysis was performed to determine the ratio of elastic fiber staining to tissue area. The deep purple areas, corresponding to elastic fibers, were identified using the Color Deconvolution plugin in ImageJ (with the Vector set to H&E 2). ROIs containing elastic fibers, excluding cholesterol plaques, were delineated into 4 to 6 sections. A uniform threshold ranging from 1 to 200 was applied to calculate the area percentage of elastic fibers in ROI.

### Immunohistochemistry (IHC)

For IHC analysis, paraffin-embedded slides (3-4 μm thick) were immunostained for CD3, CD38, CD45, CD68, MMP-9, F4/80 and α-actin. Deparaffinized sections were treated with a peroxidase-blocking solution for 10 min to quench endogenous peroxidase activity. Following rinses with PBS, the slides were immersed in pre-heated 1 mmol Tris-EDTA buffer (pH 9.0) for 15 min, then incubated at room temperature (RT) for an additional 15 min. After washing with PBS, the slides were incubated with 5% BSA for 20 min to block non-specific binding. Primary antibodies were applied and incubated at 4 °C overnight. After PBS rinses, HRP-conjugated secondary antibodies (WAS12011; World Advanced Science, China) were applied for 30 min at 37 °C. Following PBS rinses, the slides were incubated with 3,3'-Diaminobenzidine (DAB) solution (BP0770; DAKO, Denmark) and monitored under a microscope. Slides were then rinsed, counterstained with hematoxylin for 30 s at RT, dehydrated, and mounted with neutral balsam for scanning (KF-DPS-120; Kfbio, China).

The original image data were divided into four approximately equal parts with minimal overlap and blank space, each accounting for less than 20%. Using the ImageJ IHC profiler plugin, the percentage of positively stained areas was quantified. Scores from high positive, positive, and low positive areas were multiplied by factors of 3, 2, and 1, respectively, and summed for analysis.

### Live-cell staining and imaging

PDO were rinsed twice using pre-warmed (37 °C) PBS and subsequently fragmented with spring scissors. In accordance with the standard protocol outlined in the Calcein/PI Cell Viability/Cytotoxicity Assay Kit (C2015M; Beyotime, China), the minced organoid fragments were stained using a shaker at 37 °C and 90 rpm for 45 min. After rinsing twice with pre-warmed (37 °C) PBS, an appropriate amount of antifading mounting medium (S2100; Solarbio, China) was added, followed by transferring the fragments to a 96-well plate (CHIMNEY WELL, µCLEAR^®^, WHITE, CELLSTAR^®^, REF 655098; Greiner Bio-One, Germany), with a 50 μL aliquot of the suspension per well. The organoid fragments were then examined under a fluorescence microscope (DMi8; Leica, Germany).

Alternatively, the initial step can be accomplished using an enzymatic cocktail to digest the extracellular matrix, thereby releasing individual cells and potentially enhancing staining outcomes. The digestion mixture comprises Collagenase Type I (5%, 150 mg/mL) (17100-017; Gibco, USA), DNase I (1%, 10 mg/mL) (A510099-0001; Sangon Biotech, China), Elastase (1%, 200 U/mL) (abs47014929; Absin, China), and Hyaluronidase (0.4%, 2 mg/mL), each dissolved in Trypsin (0.25%, 15050057; Gibco, USA). The PDO were incubated with this mixture for 1 h, rinsed twice with PBS, and then centrifuged at 500x g to pellet the cells before proceeding with the staining procedure.

The image data were converted to 8-bit grayscale in ImageJ, followed by background subtraction with a rolling ball radius of 50 and thresholding set between 10 and 128. Measurements were taken for the mean fluorescence intensity of both the red and green channels. The ratio of green to red fluorescence intensity was then calculated. These values were normalized to the arithmetic mean of the green/red fluorescence intensity on Day 1 to determine the fold change.

### Organoid viability: alamarBlueTM assay

Following the protocol of the alamarBlue™ HS Cell Viability Reagent (Invitrogen A50100; Thermo Fisher Scientific, USA), the manipulation procedure for AAA PDO was optimized. PDO were placed into individual wells of a 24-well plate (COSTAR^®^ 3524; Corning, USA) and cultured in 1 mL of AAA organoid culture medium with 5% reagent, with a subsequent incubation for 20 h. Afterwards, the supernatant was collected, centrifuged at 3000x g for 5 min, and transferred to a 96-well plate (CHIMNEY WELL, µCLEAR^®^, WHITE, CELLSTAR^®^, REF 655098; Greiner Bio-One, Germany). The fluorescence intensity was measured using a microplate reader (Spark; TECAN, Switzerland) with excitation and emission wavelengths set at 560 nm and 595 nm, respectively. After the test, the PDO were rinsed three times with PBS and cultured in 1 mL of AAA organoid culture medium, and this medium was reused in subsequent cultivation periods. After a 2-3 days' interval, the subsequent round of testing could be initiated to monitor the activity changes of the same sample over time.

### Drug treatment for AAA PDO

On the third day of cultivation, PDO underwent drug treatment. Upon transfer to a 24-well plate (COSTAR^®^ 3524; Corning, USA), 1 mL of fresh medium containing 10 μL of the test drug solution was added to each well. The solvent of drug solution was PBS with 1% DMSO, and the solutes included 1 μM Metformin (S5958; Selleckchem, USA), 1 μM RU.521 (HY-114180; MedChemExpress, USA), 1 μM STING-IN-2 (HY-138682; MedChemExpress, USA), and solvent control, respectively. After a 48 h incubation at 37 °C with 5% CO_2_, the supernatants and PDO were collected separately and stored at -80 °C.

### Proteomics analysis

After drug treatment, the supernatants were concentrated and underwent quality control. From each sample, 15 μg of protein was extracted for separation by SDS-PAGE. Then, proteins were digested using the Filter Aided Sample Preparation (FASP) method. The peptides were further processed and subjected to chromatographic separation using the Vanquish Neo UHPLC system (Thermo Fisher Scientific), followed by Data-Independent Acquisition (DIA) mass spectrometry analysis with an Astral high-resolution mass spectrometer. The aforementioned assays and subsequent bioinformatics analysis were performed by APPLIED PROTEIN TECHNOLOGY (APTBIO) Co., Ltd, Shanghai, China, in accordance with their standard operating procedures.

### Statistical analysis

Data were collected from at least three replicates, and quantitative results are expressed as mean ± standard deviation. Statistical analysis was conducted using GraphPad Prism version 9.0.0 (GraphPad Software, USA). Student's t-test was applied for pairwise comparisons to determine significance. For comparisons involving more than two groups, one-way ANOVA was employed. A p-value less than 0.05 was considered statistically significant. Statistical significance is indicated as: *p < 0.05, **p < 0.01, and ***p < 0.001. Error bars represent the standard deviation of the mean.

## Supplementary Material

Supplementary methods and figures.

## Figures and Tables

**Table 1:** Collection of patient information

Patient No.	Gender	Age	Others
P01	Male	68	Smoking history 30 years
P02	Male	66	Smoking history > 30 years
P03	Male	66	Smoking history > 30 years
P04	Male	66	Smoking history 30 years; Coronary heart disease
P05	Male	73	Smoking history 50 years; Hypertension 20 years
P06	Male	61	N.A.
P07	Male	64	Smoking history 20 years; Coronary heart disease; Hypertension
P08	Male	67	Smoking history > 30 years; Hypertension
P09	Male	67	N.A.
P10	Male	73	Smoking history 30 years; Coronary heart disease; Hypertension
P11	Male	68	Smoking history > 40 years; Hypertension > 20 years
