# Supplementary material for: The first abdominal aortic aneurysm organoid model replicates complex microenvironment for in vitro disease study
Source: Theranostics. 2025 Aug 16;15(17):9029–46. doi: 10.7150/thno.118193 (PMC12439346; doi:10.7150/thno.118193)
Supplement: Supplementary file 1 — Supplementary methods and figures. [file thnov15p9029s1.pdf]

Supplemental Methods

Organoids Size Calculation and Analysis

The organoids were imaged routinely, and their sizes were quantified using ImageJ. A dissecting microscope (YZ38; Shanghai YueHe Biotech, China) was used to image each organoid. Given the atypical non-spheroidal shape of the organoids and overlaps in the culture dish, manual ROI (region of interest) counting was needed. The organoids' shape, determined by the tissue processing technique, was often polygonal or rectangular, making ImageJ's particle analysis function unsuitable for the area analysis. Therefore, the organoid's perimeter was manually traced in ImageJ for size analysis.

Fixation, Embedding and Sectioning

Samples were fixed in 4% PFA (BL539A; Biosharp, China) in PBS for 48 h. They were then dehydrated in 10%, 20%, and 30% sucrose (A15583.0E; Thermo Fisher Scientific, USA) in PBS for 1 h, 2 h, and overnight, respectively. After embedding in Tissue-Tek® O.C.T. Compound (4583; Sakura Finetek, Japan), samples were frozen in liquid nitrogen and cryosectioned at -20 °C (Cryostat CM1950; Leica, Germany), with sections cut perpendicular to the vessel's lumen to expose the stratified layers of the aortic wall. Alternatively, some samples were processed for paraffin embedding: after removing the fixation reagent, samples were embedded in 2% molten agarose, dehydrated (TP1020; Leica, Germany) through an ethanol series (75% for 4 h, 85% for 2 h, 90% for 2 h, 95% for 1 h, 100% for 30 min × 3), cleared in xylene (5-10 min × 2), infiltrated in 65 °C molten paraffin for 1 h × 3, and embedded using an embedding machine (KD-BM IV; KEDEE, China). Paraffin blocks were trimmed (RM2016; Leica, Germany) after cooling at - 20°C.

H&E Staining

Cryosectioned slides were fixed with 4% PFA in PBS for 10 min, rinsed in water, and stained with Hematoxylin and Eosin (H&E) (C0105S; Beyotime, China). The staining process included 10 min of hematoxylin application, 10 min of water rinsing, 5 s of differentiation with acid alcohol (C0163M; Beyotime, China), another 10 min of water rinsing, and 30 s of counterstaining with eosin. The slides were then dehydrated, mounted with neutral balsam (Type D, G8593; Solarbio, China), and examined using a standard microscope (YI21; Shanghai YueHe Biotech, China). For paraffin sections, an additional deparaffinization step was required before staining: the slides were immersed in xylene for 20 min (twice), 100% ethanol for 5 min (twice), and 75% ethanol for 5 min, followed by water rinsing. After staining, the sections were dehydrated with 100% ethanol, clarified with xylene for 5 min, mounted with neutral balsam, and prepared for scanning (KF-DPS-120; Kfbio, China).

For quantitative analysis based on H&E staining, ImageJ was used to calculate the ratio of cell nuclei number per section area. Images were processed with the Color Deconvolution plugin using H&E Vectors to separate Hematoxylin-stained components. Then, a threshold of 1 - 180 was applied, fine-tuning as necessary to clearly display cell nuclei without severe overlap and minimal background noise. Six non - overlapping ROIs, each 600 μm^2, were selected. Analyze Particles function was applied to each ROI with a size range of 5 - Infinity μm^2 for counting.

Verhoeff-van Gieson Elastic Staining

Cryosectioned slides were fixed in 4% PFA in PBS for 10 min, rinsed in water for 10 min, and stained with a mixture of 5% alcoholic hematoxylin (HE-013-100mL; BIOISCO, China), 10%

ferric chloride (Iron(III) chloride, anhydrous, A600454-0500; Sangon Biotech, China), and Lugol's Iodine Solution (MM1049-100mL; MKBio, China) in a 1:1:0.5 ratio for 15 min. After rinsing, slides were differentiated with 2% ferric chloride, rinsed again, soaked in 5% sodium thiosulfate (217263-5G; Sigma-Aldrich, USA), rinsed, and stained with Van Gieson's Stain (MM1032; MKBio, China) for 30 s. Finally, slides were dehydrated with 100% alcohol and mounted using neutral balsam for examination under a standard microscope.

Quantitative analysis was performed to determine the ratio of elastic fiber staining to tissue area. The deep purple areas, corresponding to elastic fibers, were identified using the Color Deconvolution plugin in ImageJ (with the Vector set to H&E 2). ROIs containing elastic fibers, excluding cholesterol plaques, were delineated into 4 to 6 sections. A uniform threshold ranging from 1 to 200 was applied to calculate the area percentage of elastic fibers in ROI.

Immunohistochemistry (IHC)

Paraffin-embedded slides (3-4 μm) were immunostained for CD3, CD38, CD45, CD68, MMP-9, F4/80 and α-actin. Deparaffinized sections were treated with a peroxidase-blocking solution for 10 min, immersed in pre-heated 1 mmol Tris-EDTA buffer (pH 9.0) for 15 min, then incubated at room temperature (RT) for 15 min. After washing with PBS, the slides were incubated with 5% BSA for 20 min. Primary antibodies were applied and incubated at 4°C overnight. After PBS rinses, HRP-conjugated secondary antibodies (WAS12011; World Advanced Science, China) were applied for 30 min at 37 °C. The slides were then incubated with DAB solution (BP0770; DAKO, Denmark) and monitored under a microscope. Finally, slides were rinsed, counterstained with hematoxylin for 30 s at RT, dehydrated, and mounted with neutral balsam for scanning (KF-DPS-120; Kfbio, China).

The original image data were divided into four parts with minimal overlap and blank space (< 20% each). Using the ImageJ IHC profiler plugin, the percentage of positively stained areas was quantified. Scores from high positive, positive, and low positive areas were multiplied by 3, 2, and 1, respectively, and summed for analysis.

ECM digestion (optional step for Live-Cell Imaging)

Alternatively, an enzymatic cocktail can be used to digest ECM, releasing individual cells and potentially enhancing staining. The digestion mixture includes Collagenase Type I (5%, 150 mg/mL) (17100 - 017; Gibco, USA), DNase I (1%, 10 mg/mL) (A510099 - 0001; Sangon Biotech, China), Elastase (1%, 200 U/mL) (abs47014929; Absin, China), and Hyaluronidase (0.4%, 2 mg/mL), all dissolved in Trypsin (0.25%, 15050057; Gibco, USA). Organoids were incubated with this mixture for one h, rinsed twice with PBS, centrifuged at 500x g and then subjected to Calcein AM/PI staining.

Organoid Viability: alamarBlueTM Assay

The alamarBlue™ HS assay, using a non-fluorescent blue dye, was directly incorporated into the culture medium and incubated for 20 h, converting to a strong red fluorescent product by viable cells. Following the protocol of the alamarBlue™ HS Cell Viability Reagent (Invitrogen A50100; Thermo Fisher Scientific, USA), the manipulation procedure for AAA organoids was optimized. Organoids were placed into individual wells of a 24-well plate (COSTAR® 3524; Corning, USA) and cultured in 1 mL of AAA organoid culture medium with 5% reagent, with a subsequent incubation for 20 h. Afterwards, the supernatant was collected, centrifuged at 3000x g for 5 min, and transferred to a 96-well plate (CHIMNEY WELL, μCLEAR®, WHITE, CELLSTAR®, REF

655098; Greiner Bio-One, Germany). The fluorescence intensity was measured using a microplate reader (Spark; TECAN, Switzerland) with excitation and emission wavelengths set at 560 nm and 595 nm, respectively. Subsequently, the organoids were rinsed three times with PBS and cultured in 1 mL fresh medium. After 2-3 days, repeat testing to monitor sample activity changes over time.

Supplemental Figure

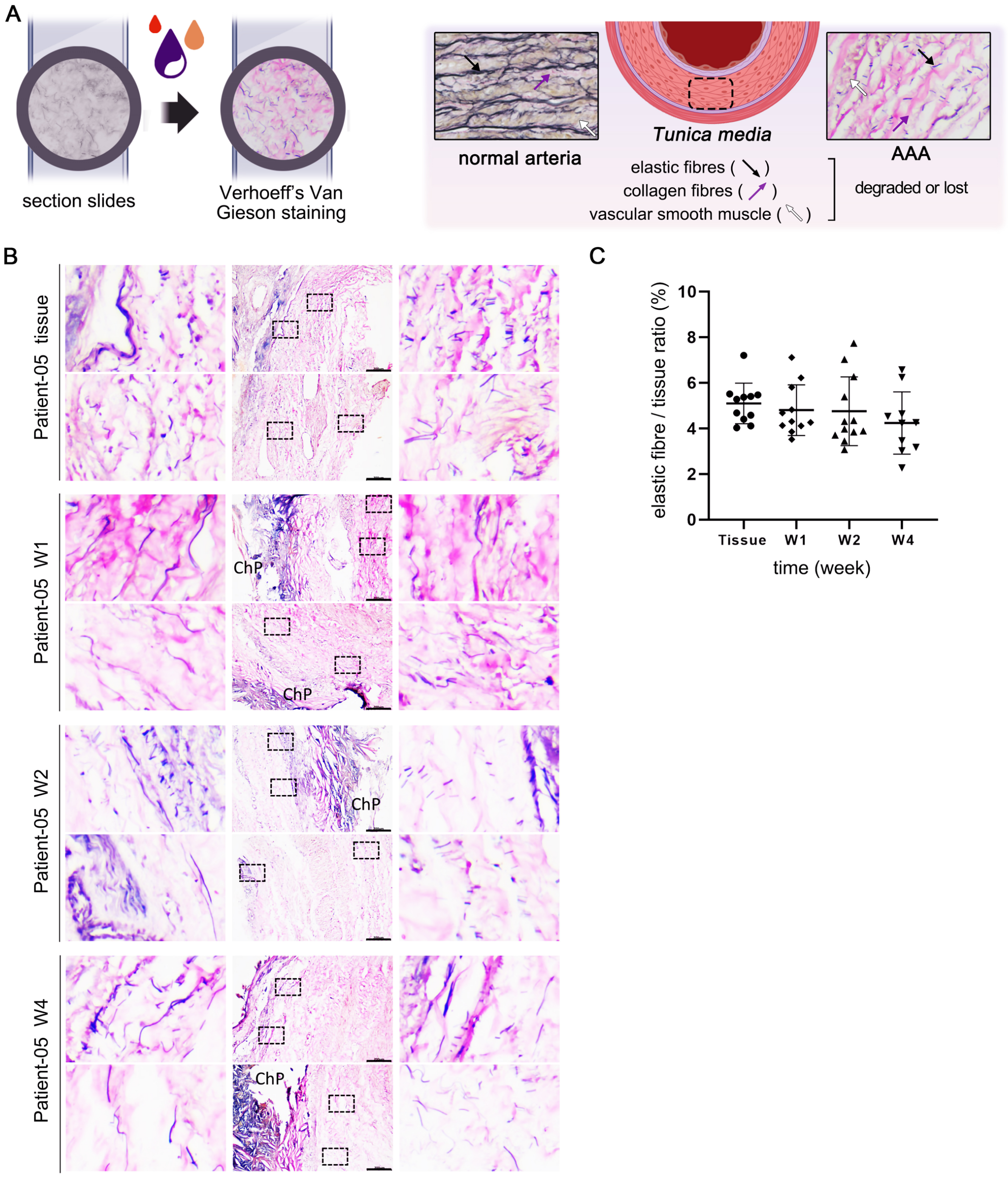
A
section slides
Verhoeff's Van Gieson staining
normal arteria
Tunica media
AAA
elastic fibres ( )
collagen fibres ( )
vascular smooth muscle ( )
degraded or lost
B
Patient-05 tissue
Patient-05 W1
Patient-05 W2
Patient-05 W4
ChP
C
elastic fibre / tissue ratio (%)
10
8
6
4
2
0
Tissue
W1
W2
W4
time (week)

97

FigureS1 Elastic Staining and Analysis in AAA PDO. (A) Schematic diagram illustrating the Verhoeff-van Gieson elastic staining procedure. Elastic staining image of normal human abdominal aortic tissue is obtained from John A. Curci [87] and modified with BioRender.com and Adobe PhotoShop 2024. (B) Cryosection staining images from Patient 05. Scale Bar and longer side of inset = 200 μm. ChP = Cholesterol Plaque. (C) Quantitative analysis of the stained elastic fiber. The deep purple areas were extracted using the Color Deconvolution 2 plugin in ImageJ (with Vector set to H&E 2). The region containing elastic fibers (except cholesterol plaques) were divided into 4-6 ROIs, and a consistent threshold (1-200) was applied to calculate the ratio of the area occupied by elastic fibers to the total ROI area. Data are represented as the mean ± SEM, with individual data points displayed. Illustration and statistical analysis were performed using GraphPad 9.0.

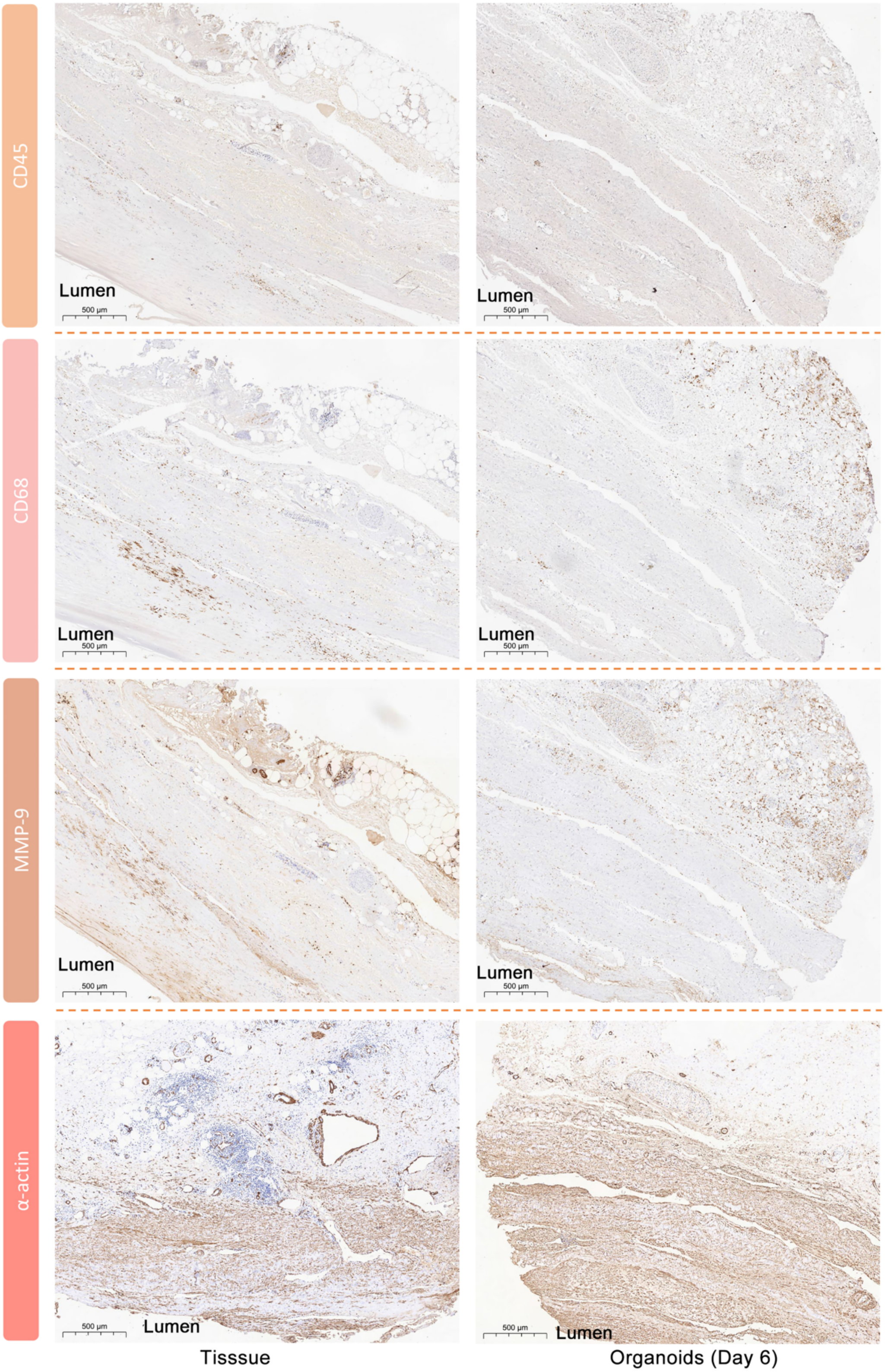

109

FigureS2 IHC images of related markers at low magnification. scale bar = 500 μm.
